# Correlation of maximal inspiratory pressure to transdiaphragmatic twitch pressure in intensive care unit patients

**DOI:** 10.1186/s13054-016-1247-z

**Published:** 2016-03-23

**Authors:** Gerald S. Supinski, Phillip Westgate, Leigh A. Callahan

**Affiliations:** Department of Internal Medicine, Division of Pulmonary, Critical Care and Sleep Medicine, University of Kentucky, 740 South Limestone Room L-543, Lexington, KY 40536-0284 USA; Department of Biostatistics, University of Kentucky, 725 Rose Street, Lexington, KY 40536-0082 USA

## Abstract

**Background:**

Respiratory muscle weakness contributes to respiratory failure in ICU patients. Unfortunately, assessment of weakness is difficult since the most objective test, transdiaphragmatic pressure in response to phrenic nerve stimulation (PdiTw), is difficult to perform. While most clinicians utilize maximum inspiratory pressure (Pimax) to assess strength, the relationship of this index to PdiTw has not been evaluated in a large ICU population. The purpose of the present study was to assess both PdiTw and Pimax in ICU patients to determine how these indices correlate with each other, what factors influence these indices, and how well these indices predict outcomes.

**Methods:**

Studies were performed on adult mechanically ventilated patients in the University of Kentucky MICU (n = 60). We assessed PdiTw by measuring transdiaphragmatic pressure (Pdi) in response to bilateral twitch stimulation of the phrenic nerves using dual magnetic stimulators (Magstim 200). Pimax was determined by measuring airway pressure during a 30-second inspiratory occlusion. We also assessed the twitch and maximum force generation for diaphragms excised from control and septic mice.

**Results:**

Both Pimax and PdiTw measurements were profoundly reduced for mechanically ventilated MICU patients when compared to normal reference values, e.g., Pimax averaged 56 % of the predicted value for normal subjects. For the ICU population as a whole, PdiTw and Pimax values correlated with each other (r^2^ = 0.373, *p* < 0.001), but there was wide scatter and, as a result, PdiTw could not be reliably calculated from Pimax levels for individual subjects. Infection selectively reduced low-frequency force generation more than high-frequency force generation for both our mouse experiments (comparing muscle twitch to 150 Hz tetanic force) and for MICU patients (comparing PdiTw to Pimax). This effect of infection may contribute to scatter in the PdiTw to Pimax relationship. We also found that both PdiTw and Pimax were significantly correlated with both patient survival and the duration of mechanical ventilation, albeit statistically, PdiTw was the better predictor.

**Conclusions:**

While more difficult to measure, the PdiTw is a better predictor of outcomes in mechanically ventilated MICU patients than the Pimax. Nevertheless, for some clinical applications, the Pimax determination is a reasonable alternative.

**Electronic supplementary material:**

The online version of this article (doi:10.1186/s13054-016-1247-z) contains supplementary material, which is available to authorized users.

## Background

Recent work indicates that severe respiratory muscle weakness is commonly present in mechanically ventilated medical intensive care unit (MICU) patients and may be a major determinant of the duration of respiratory failure in this patient population [1–5]. Objective measurement of respiratory muscle strength in these patients is therefore important clinically, since this assessment can provide both diagnostic and prognostic information and can be used to assess the effects of therapeutic agents designed to augment muscle function. Unfortunately, accurate assessment of the degree of respiratory muscle weakness in MICU patients is difficult since the most objective diagnostic test, determination of transdiaphragmatic pressure generation in response to bilateral phrenic nerve stimulation (PdiTw), is technically difficult to perform [6]. As an alternative, clinicians can assess maximum inspiratory pressure (Pimax or MIP) as an index of respiratory muscle strength, but some reports have found this latter technique to be unreliable in MICU patients [7]. Most importantly, there has never been a large study in which both PdiTw and Pimax were measured and compared to each other in a diagnostically broad-based group of mechanically ventilated MICU patients.

The purpose of the present study, therefore, was to assess both PdiTw and Pimax in a large cross section of intensive care unit (ICU) patients and determine how these indices correlate with each other and with other clinical parameters. We sought to determine: (a) the degree to which Pimax measurements could be used to predict PdiTw determinations, (b) if there were clinical factors which systematically altered the relationship between PdiTw and Pimax, and (c) which index (PdiTw or Pimax) was the best predictor of patient outcomes (mortality, duration of mechanical ventilation).

The database upon which the information in the present report is based was previously used to generate an article that evaluated the role of infection in eliciting weakness in MICU patients [4]. This previous article, however, did not report or evaluate Pimax levels. The current manuscript extends the work in this previous publication by determining the relationship of Pimax to PdiTw and assessing the value of the Pimax as a predictor of patient outcomes.

## Methods

### Patient selection

Approval to conduct this research was obtained from the University of Kentucky Institutional Review Board (IRB). Consent for inclusion in these studies was obtained from all subjects and/or their surrogates. Inclusion into the study was considered for all adult patients requiring mechanical ventilation for more than 24 hours for respiratory failure in one of the University of Kentucky adult MICUs. Subjects were included regardless of sex, race, or age. Subjects were excluded: (a) if the physician caring for the patient determined that the patient was too unstable to tolerate these measurements, or if the patient (b) was receiving high-dose pressors (more than 15 mcg/min of norepinephrine or more than 15 mg/kg/min of dopamine), (c) required > 80 % fraction of inspired oxygen (FiO_2_) or > 15 cm water (H_2_O) of positive end-expiratory pressure (PEEP), (d) had a cardiac pacemaker or implanted defibrillator, (e) received neuromuscular blocking agents within the 48 hours preceding testing, (f) had a history of a preexisting neuromuscular disease, (g) recent variceal bleeding, (h) was pregnant, (i) was incarcerated, or (j) was institutionalized.

### Study protocol

After consent was obtained, the following parameters were recorded: (a) ventilator triggering, (c) respiratory static system compliance and airway resistance, (c) diaphragm strength by measuring PdiTw (transdiaphragmatic twitch pressure), (d) global inspiratory muscle strength by measuring Pimax (maximum inspiratory pressure), and (e) clinical data and outcomes, including mortality and additional days required for continued mechanical ventilation until successful extubation.

A total of 60 patients were studied. In three of these, it was not possible to attain supramaximal phrenic nerve activation, precluding adequate measurement of PdiTw. These patients were therefore excluded; measurements obtained on the remaining 57 patients were used for data analysis.

Details regarding “Assessment of mechanical ventilator triggering”, “Measurement of respiratory system static compliance and airway resistance”, “Determination of transdiaphragmatic twitch pressure generation (PdiTw)”, and “Determination of maximum inspiratory pressure (Pimax)” are provided in Additional file 1.

### Clinical parameters

Clinical parameters were collected as close as possible to the time of determination of PdiTw and Pimax. The following were assessed: age, gender, diagnoses, the presence of positive cultures for infectious agents, antibiotic regimen, vital signs, duration of mechanical ventilation prior to PdiTw measurement, mechanical ventilation mode, FiO_2_, tidal volume and rate, percentage of patient-triggered breaths, and most recent arterial blood gas values. The Sequential Organ Failure Assessment (SOFA) score and Charlson Comorbidity Index were recorded for each subject [8, 9]. We also determined the time required to wean patients from mechanical ventilation after measurement of PdiTw and recorded if the patient died or survived their MICU stay.

### Animal studies

Studies were performed on 10 mice to determine how infection alters the relationship between twitch and maximum diaphragmatic specific force generation. These experiments were approved by the University of Kentucky Institutional Animal Care and Use Committee (IACUC). Five mice were given saline intraperitoneally (0.5 ml) and the remaining five were given endotoxin (i.e., 60,000,000 units/kg lipopolysaccharide (LPS) from E. coli, 55:B5, Sigma-Aldrich, St. Louis, MO, USA in 0.5 ml saline). Animals were sacrificed at 24 hours after injections and the diaphragm excised. Diaphragm strips were then dissected and used to assess diaphragm force generation in vitro as described in detail in Additional file 1 and as described in previous publications [10, 11].

### Statistics

To compare the Pimax measurements obtained in our patients to “normal” values for healthy men and women, we used the regression equations developed by Enright et al. [12]. Specifically, normal Pimax levels for women = (0.133 Wt) - (0.805 Age) + 96 and normal Pimax levels for men = (0.131 Wt) - (1.27 Age) + 153, where Wt is patient weight [12]. We therefore used these equations to calculate the Pimax levels our patients would have achieved had they been normal and compared these normal values to the actual Pimax levels obtained during our study.

Whenever data were normally distributed and variances were similar, parametric tests were used to compare groups. When these conditions were not met, nonparametric tests were used to make comparisons. Data analyzed using parametric tests are presented as mean ± 1 standard error of the mean. Data analyzed using nonparametric tests are presented as median ± confidence intervals.

Comparison of actual Pimax to reference equation predicted levels of Pimax was made using a paired *t* test. The relationship of Pimax to PdiTw was analyzed using linear regression analysis. Comparison of PdiTw, Pimax, and Pimax/PdiTw ratios between noninfected and infected patients was performed using unpaired *t* tests. Comparison of twitch, 150 Hz force, and the 150 Hz/twitch force ratios between control and LPS (endotoxin)-treated animals was carried out using unpaired *t* tests. Assessment of the relationships between PdiTw, Pimax or PesoTw (esophageal twitch pressure) and either mortality or duration of mechanical ventilation was accomplished using dynamic curve analysis employing the SigmaStat statistical package (Jandel Scientific Software, San Rafael, CA, USA). In all cases, a *p* value less than 0.05 was taken as evidence of statistical significance.

## Results

### Comparison of Pimax to PdiTw measurements

**Fig. 1:**
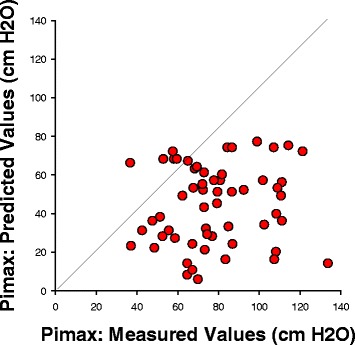
This figure displays Pimax values for the cohort of patients included in this study, with predicted values on the x axis (calculated based on published data for normal, healthy adults) and measured values on the y axis. The figure also includes a line of identity; points above this line represent patients whose measured Pimax was greater than predicted, while points below this line represent patients with Pimax lower than predicted. Only four patients had points above the line, with the remaining 53 patients being weaker than predicted. *Pimax* maximum inspiratory pressure

A total of 60 patients were recruited into the study. In three of these individuals, technically adequate measurements of PdiTw could not be performed. These three patients were excluded from the study, and data from the remaining 57 patients was used for analysis. Pimax averaged a value of 44.3 ± 2.7 cm H_2_O for these 57 patients. As shown in Fig. 1, measured Pimax was appreciably lower than predicted Pimax in all but four patients. On average, measured Pimax was 56 % of the value expected for healthy age-, sex- and weight-matched controls (i.e., 78.5 ± 2.9 cm H_2_O, *p* < 0.001 for comparison of actual Pimax to predicted Pimax) [12]. The coefficient of variation for Pimax determinations in our study averaged 7.46 ± 0.01 %.

**Fig. 2:**
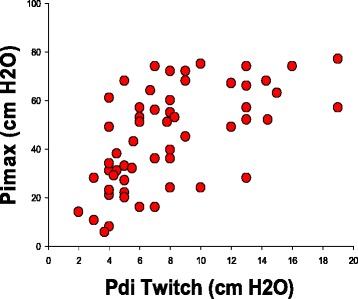
The panel plots Pimax values (y axis) against PdiTw values (x axis) with each point representing an individual patient in our study cohort. *PdiTw* transdiaphragmatic twitch pressure in response to bilateral magnetic stimulation of the phrenic nerves, *Pimax* maximum inspiratory pressure

Figure 2 displays Pimax versus PdiTw measurements for individual patients. There was a fair correlation between these two indices, with Pimax = 3.0 (PdiTw) + 20.7 by linear regression analysis, with an r^2^ of 0.373, *p* < 0.001 for this relationship. While Pimax was statistically correlated with PdiTw for our patient population as a whole, there was substantial scatter in this relationship such that in individual patients PdiTw measurements could not be reliably estimated from Pimax determinations. For example, one individual with a Pimax of 57 cm H_2_O had a PdiTw of 6 cm H_2_O, while another patient with the same Pimax of 57 had a PdiTw of 20 cm H_2_O.

### Effect of infection and respiratory drive on the relationship between Pimax and Pdi twitch

**Fig. 3:**
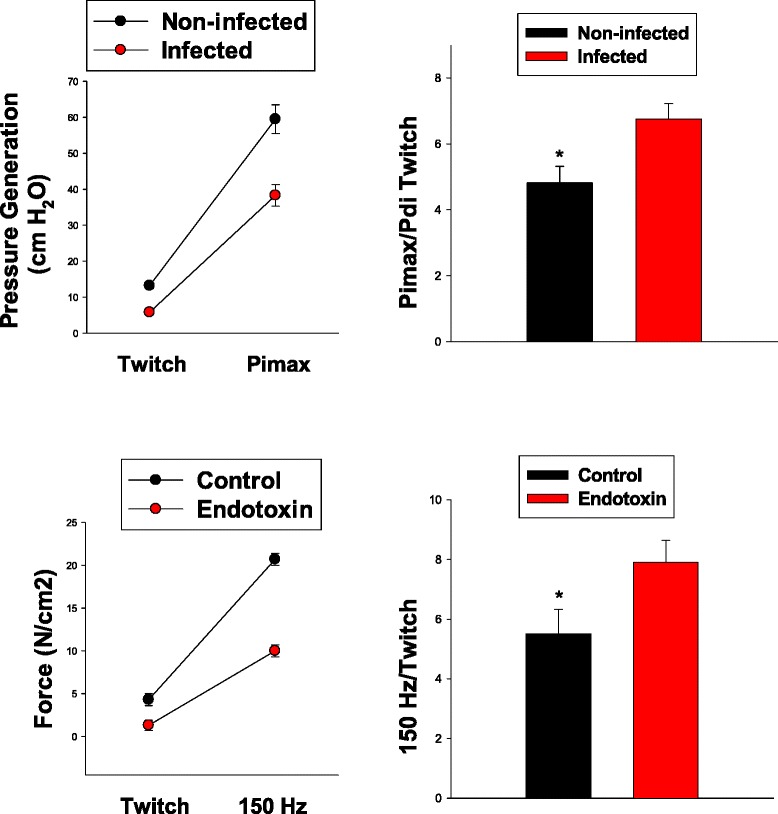
The *top panels* in this figure represent data from the patient cohort included in this study and the *bottom panels* represent data from animal studies comparing control animals to animals treated with endotoxin (i.e., LPS, lipopolysaccharide). The *upper left panel* compares PdiTw and Pimax values for noninfected patients (*black symbols*) to infected patients (*red symbols*). Both PdiTw and Pimax values for infected patients were significantly lower than values for noninfected patients, but the relative infection-induced reductions were substantially greater for twitch measurements than for Pimax levels. As a result, the Pimax/twitch ratio for noninfected patients was significantly lower than for infected patients (*right top panel*, ^*^indicates a significant difference, *p* < 0.001). The same pattern of findings was observed for animal studies, with both diaphragm twitch and maximal force (i.e., force in response to150 Hz) for endotoxin-treated animals significantly lower than for control animals (*left lower panel*). In addition, the 150 Hz/twitch force ratio was significantly lower for control than for endotoxin-treated animals (*p* = 0.042). *PdiTw* transdiaphragmatic twitch pressure in response to bilateral magnetic stimulation of the phrenic nerves, *Pimax* maximum inspiratory pressure

One possible explanation for the substantial scatter in the relationship between Pimax and PdiTw could be that both measurements are technically difficult to make, with a large inherent variability in these determinations. The fact that the coefficients of variation for Pimax and PdiTw measurements in individual subjects were quite small (coefficient of variability of 7 % for Pimax and 7 % for PdiTw) suggests, however, that this is not the only factor accounting for variation in these parameters. One such factor may be the presence of infection. The patients in this dataset were classified as infected or noninfected based on culture results, attending physician diagnoses, and the use of antibiotics as previously described [4]. Both Pimax and PdiTw levels were significantly lower for infected as compared to noninfected patients (Fig. 3, *top left panel*, *p* < 0.001 for comparison of Pimax and *p* = 0.019 for comparison of PdiTw between infected and noninfected groups). The relative magnitude of infection-induced reductions in Pimax and PdiTw differed, however, with a significantly greater fall in PdiTw due to infection than the reduction in Pimax. This is best observed by evaluating the Pimax/PdiTw ratio. As shown in Fig. 3, *top right panel*, this ratio was significantly lower for noninfected patients than infected patients (*p* < 0.001 for comparison of this ratio between infected and noninfected groups).

To further evaluate this phenomenon, we used an animal model of infection (endotoxin administration to mice) and determined the effect of endotoxin on the force-frequency relationship of the diaphragm. We took the in vitro diaphragm force generated in response to a single electrical impulse for these animals as an index of the in vivo PdiTw and we took the diaphragm force generated in response to a 150 Hz train of impulses as an index of the maximal in vivo respiratory pressure generation, i.e., the Pimax. As shown in Fig. 3, *bottom left and right panels*, endotoxin administration to mice altered twitch and 150 Hz diaphragm force output in a manner analogous, respectively, to the effects of infection on PdiTw and Pimax measurements in our mechanically ventilated subjects. Specifically, endotoxin administration had a greater effect to reduce twitch force than 150 Hz force, and, as a result the 150 Hz/twitch force ratio was lower for control animals and significantly higher for endotoxin-treated animals (Fig. 3, *bottom right panel*).

**Fig. 4:**
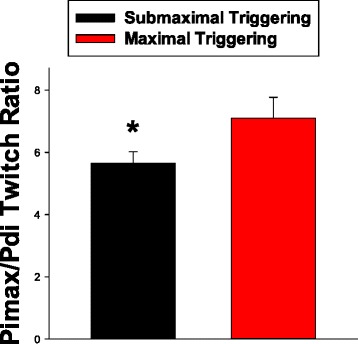
This figure displays and compares Pimax/PdiTw ratios for the patients in our study who were maximally triggering ventilator breaths (i.e., patients that triggered all mechanical ventilator breaths at the time of assessment, *red bar*) against the Pimax/PdiTw ratios for patients who only partially initiated breaths, with some or all breaths initiated by the mechanical ventilator in the absence of patient effort (*black bar*). The Pimax/PdiTw ratio was significantly higher for patients who triggered all breaths than for patients with submaximal triggering (*p* = 0.044, ^*^indicates a significant difference from the other group). *PdiTw* transdiaphragmatic twitch pressure in response to bilateral magnetic stimulation of the phrenic nerves, *Pimax* maximum inspiratory pressure

Patients with less respiratory drive (e.g., less lung disease, more sedation) might be expected to generate less effort with Pimax measurements, with the result that low drive levels could reduce Pimax and the Pimax/PdiTw ratio. Using ventilator triggering as an index of respiratory drive, we found that patients who triggered all breaths had a significantly higher Pimax/PdiTw ratio (Fig. 4, *p* < 0.01). This finding is consistent with the concept that patients with lower levels of respiratory drive, who would be expected to have less triggering, generate lower Pimax levels for a given PdiTw than patients with higher levels of respiratory drive and higher rates of ventilator triggering.

### Relationship of Pimax and PdiTw to patient outcomes

**Fig. 5:**
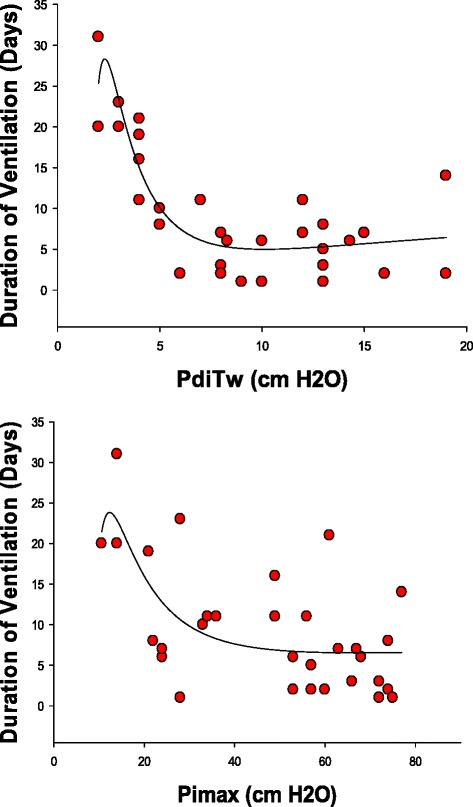
The *top panel* presents the duration of mechanical ventilation (y axis) as a function of the PdiTw (x axis). The *bottom panel* presents the duration of mechanical ventilation (y axis) as a function of the Pimax (x axis). Regression lines represent best curve fits of data to inverse third order exponential equations, with equation parameters and statistical information presented in Table 1. Both PdiTw and Pimax were significant predictors of duration of mechanical ventilation. *PdiTw* transdiaphragmatic twitch pressure in response to bilateral magnetic stimulation of the phrenic nerves, *Pimax* maximum inspiratory pressure

**Fig. 6:**
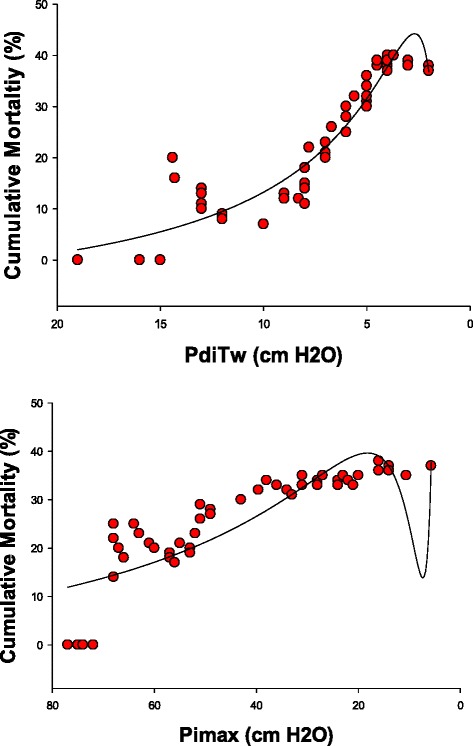
The *top panel* presents the cumulative mortality of patients (y axis) as a function of PdiTw (x axis). The *bottom panel* presents the cumulative mortality of patients (y axis) as a function of the Pimax (x axis). Regression lines represent best curve fits of data to inverse third order exponential equations, with equation parameters and statistical information presented in Table 1. Both PdiTw and Pimax were significant predictors of cumulative mortality. *PdiTw* transdiaphragmatic twitch pressure in response to bilateral magnetic stimulation of the phrenic nerves, *Pimax* maximum inspiratory pressure

Figures 5 and 6 present the relationship between the PdiTw and Pimax measurements and patient mortality and duration of mechanical ventilation, We found that both PdiTw and Pimax were statistically correlated with duration of mechanical ventilation, with dynamic curve analysis indicating that the best curve fit for both parameters was achieved using inverse exponential equations (parameters for these analysis are presented in Table 1). As is evident by inspection of Fig. 5 and Table 1, PdiTw was the better predictor of weaning duration. Patients having PdiTw levels more than 10 cm H_2_O weaned from mechanical ventilation in an average of 5.5 days (*top panel*, Fig. 5). In contrast, as PdiTw fell below to lower levels, duration of mechanical ventilation increased substantially, increasing to greater than 10 days for all patients with PdiTw less than 4 cm H_2_O. There was greater scatter for the relationship of Pimax to duration of mechanical ventilation (*bottom panel*, Fig. 5), but there was still a statistically significant curvilinear relationship between Pimax and duration, with patients having a Pimax less negative than 25 cm H_2_O requiring more than 10 days, on average, to wean from mechanical ventilation.

**Table 1:** Statistical analysis of duration of mechanical ventilation and mortality

Outcome	Index	yo	a	b	c	R^2^	F	*p*
Duration of ventilation	PdiTw	−122	−158	988	−1236	0.75	27.1	< 0.0001
Duration of ventilation	Pimax	96	−431	17193	−119569	0.42	6.4	< 0.002
Mortality	PdiTw	−12.3	290	−325	−120	0.90	129.9	< 0.0001
Mortality	Pimax	−14.9	2325	−29779	103878	0.73	40.0	< 0.0001
Duration of ventilation	PesoTw	11.6	−102	464	−435	0.72	22.9	< 0.0001
Mortality	PesoTw	−10	4.18	487	−755	0.77	50.4	< 0.0001

All relationships represent nonlinear regression curve fits to an inverse third order exponential equation, i.e. y = yo + a/x + b/x^2^ + c/x^3^

The correlation coefficient of the equation is presented as R^2^. Also provided are the F statistic for the curve fit and the *p* value

*PdiTw* transdiaphragmatic twitch pressure in response to bilateral magnetic stimulation of the phrenic nerves

*PesoTw* esophageal twitch pressure in response to bilateral magnetic stimulation of the phrenic nerves, *Pimax* maximum inspiratory pressure

As shown in Fig. 6, both PdiTw and Pimax also were statistically significant predictors of mortality. Assessment of these relationships was again accomplished employing dynamic curve analysis using inverse exponential equations, with parameters for these constructs presented in Table 1. For PdiTw analysis (Fig. 6, *top panel*), the mortality rate remained low for strength above a PdiTw level of 10 cm H_2_O. As strength fell below a PdiTw level of 10 cm H_2_O, however, the cumulative mortality rate progressively rose, increasing to a level close to 40 %. Cumulative mortality was also statistically correlated with Pimax levels, with increasing mortality notable as Pimax fell to levels less negative than 50 cm H_2_O (Fig. 6, *bottom panel*). The shape of the mortality curves differed for these two parameters, with the PdiTw curve only demonstrating sharp increases in mortality when PdiTw fell to very low levels, but with a substantial rise in mortality with relatively modest reductions in the Pimax.

To further assess the relationship between mortality and respiratory muscle strength, we conducted a multivariate analysis to adjust for potential confounding variables, including sepsis, age, gender, respiratory system static compliance, inspiratory airway resistance, steroid usage, the severity of organ failure (SOFA score), and the Charlson Comorbidity Index. The analysis of PdiTw and these confounding variables (Table 2) found that PdiTw (x in the analysis) had a highly significant relationship to mortality (*p* = 0.0003) but none of the other variables (sepsis, age, gender, respiratory system static compliance, inspiratory airway resistance, steroid usage, the severity of organ failure (SOFA score), and the Charlson Comorbidity Index) were significantly related to mortality. When we analyzed Pimax and these confounding variables (Table 3) we found that Pimax was also significantly related to mortality while none of the other variables were statistically significant predictors. Finally, we also analyzed the subgroup of septic patients and examined the relationship of our two indices of strength and the remaining confounding variables (age, gender, respiratory system static compliance, inspiratory airway resistance, steroid usage, SOFA score, and the Charlson Comorbidity Index) to mortality (results for these subgroup analyses are included in Tables 4 and 5). Evaluation of PdiTw in the sepsis subgroup found that PdiTw was highly correlated with mortality (*p* < 0.0001), the Charlson Comorbidity Index had a good correlation with mortality (*p* = 0.01) and none of the other confounding variables had a significant correlation with mortality. Evaluation of Pimax in the sepsis subgroup found that Pimax was highly correlated with mortality (*p* < 0.0001), the SOFA score had a fair correlation with mortality (*p* = 0.02) and none of the other confounding variables significantly correlated with mortality.

**Table 2:** Full linear regression model results for cumulative mortality (%) index: x is PdiTw

Variable	Estimate	95 % Confidence interval	*p* value
Intercept	−26	(−40, −11)	0.001
1/x	403	(201, 605)	0.0003
1/x^2^	−779	(−1614, 55)	0.07
1/x^3^	423	(−579, 1425)	0.40
Sepsis			
Yes	−3.29	(−8.34, 1.77)	0.20
No	Reference		
Age	0.06	(−0.03, 0.15)	0.21
Steroids			
Yes	0.12	(−2.59, 2.83)	0.93
No	Reference		
Lung compliance	0.04	(−0.11, 0.19)	0.57
Airway resistance	0.18	(−0.01, 0.36)	0.06
Severity of Organ Failure Assessment (SOFA)	0.004	(−0.57, 0.58)	0.99
Charlson Comorbidity Score	0.45	(−0.29, 1.18)	0.23
Gender			
Female	−0.44	(−3.13, 2.25)	0.74
Male	Reference

**Table 3:** Full linear regression model results for cumulative mortality (%) index: x is Pimax

Variable	Estimate	95 % Confidence interval	*p* value
Intercept	−10	(−23, 3)	0.13
1/x	2164	(1459, 2868)	< 0.0001
1/x^2^	−27869	(−39210, −16527)	< 0.0001
1/x^3^	97262	(52929, 141594)	< 0.0001
Sepsis			
Yes	4.30	(−0.70, 9.30)	0.09
No	Reference		
Age	0.01	(−0.12, 0.15)	0.84
Steroids			
Yes	0.08	(−3.96, 4.12)	0.97
No	Reference		
Lung compliance	−0.09	(−0.31, 0.12)	0.40
Airway resistance	−0.19	(−0.46, 0.09)	0.18
Severity of Organ Failure Assessment (SOFA)	−0.28	(−1.17, 0.60)	0.52
Charlson Comorbidity Score	0.22	(−0.93, 1.37)	0.70
Gender			
Female	1.95	(−2.19, 6.09)	0.35
Male	Reference

**Table 4:** Full linear regression model results for cumulative mortality (%) index: x is PdiTw

Variable	Estimate	95 % Confidence interval	*p* value
Intercept	−40	(−53, −27)	< 0.0001
1/x	661	(488, 835)	< 0.0001
1/x^2^	−1752	(−2437, −1068)	< 0.0001
1/x^3^	1491	(700, 2283)	0.001
Age	0.003	(−0.06, 0.07)	0.91
Steroids			
Yes	1.08	(−0.71, 2.88)	0.22
No	Reference		
Lung compliance	−0.08	(−0.20, 0.04)	0.18
Airway resistance	0.002	(−0.12, 0.12)	0.98
Severity of Organ Failure Assessment (SOFA)	−0.13	(−0.49, 0.23)	0.46
Charlson Comorbidity Score	0.57	(0.13, 1.02)	0.01
Gender			
Female	0.23	(−1.45, 1.91)	0.78
Male	Reference		

Subgroup analysis includes only those patients with sepsis

**Table 5:** Full linear regression model results for cumulative mortality (%) index: x is Pimax

Variable	Estimate	95 % Confidence interval	*p* value
Intercept	2	(−8, 12)	0.72
1/x	1868	(1343, 2393)	< 0.0001
1/x^2^	−23257	(−31474, −15040)	< 0.0001
1/x^3^	79159	(47448, 110871)	< 0.0001
Age	0.02	(−0.09, 0.13)	0.70
Steroids			
Yes	1.48	(−1.76, 4.72)	0.35
No	Reference		
Lung compliance	−0.09	(−0.31, 0.14)	0.43
Airway resistance	−0.14	(−0.35, 0.08)	0.21
Severity of Organ Failure Assessment (SOFA)	−0.82	(−1.51, −0.14)	0.02
Charlson Comorbidity Score	0.56	(−0.33, 1.45)	0.20
Gender			
Female	−1.40	(−4.60, 1.79)	0.37
Male	Reference		

Subgroup analysis includes only those patients with sepsis

### Relationship of twitch esophageal pressure to outcomes

**Fig. 7:**
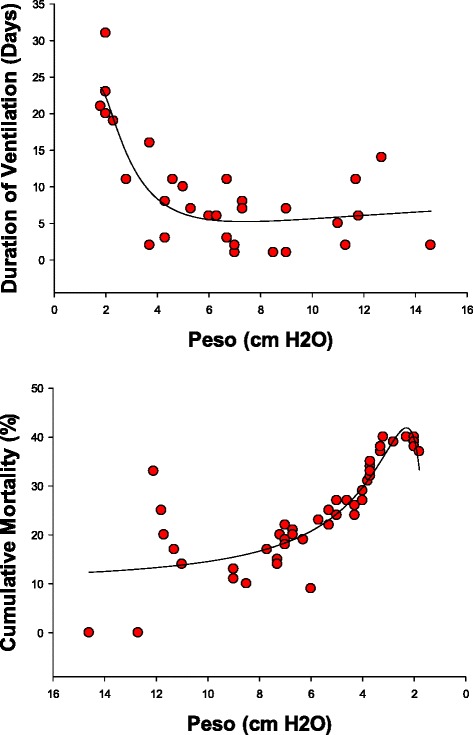
The *top panel* presents the duration of mechanical ventilation (y axis) as a function of the PesoTw (x axis). The *bottom panel* presents the cumulative mortality of patients (y axis) as a function of the PesoTw (x axis). Regression lines represent best curve fits of data to inverse third order exponential equations. PesoTw significantly predicted both the duration of mechanical ventilation and cumulative mortality. *PesoTw* esophageal pressure generated during the twitch assessment

We also examined the relationship of one of the components of the PdiTw, namely the esophageal pressure generated during the twitch assessment (PesoTw), to duration of mechanical ventilation and mortality. PesoTw was a fairly good predictor of both outcomes, with patients generating lower PesoTw having a longer duration of mechanical ventilation and a high mortality (Fig. 7). On a statistical basis, PesoTw was a somewhat weaker predictor of outcomes than PdiTw but had better F values than Pimax (see Table 1).

## Discussion

Studies performed in the past 10 years indicate that many mechanically ventilated MICU patients have severe diaphragm weakness [1–5]. Moreover, diaphragm weakness is thought to be associated with poor outcomes in this patient population, with the weakest patients requiring more prolonged mechanical ventilation and having a significantly higher mortality than patients with better diaphragm strength [1, 4]. Because of this evidence, it is speculated that mechanically ventilated patients may benefit from treatment with anabolic agents which increase skeletal muscle strength. In theory, such agents could improve patient outcomes, reducing duration of mechanical ventilation and patient mortality. Several pharmacological agents have been shown to improve skeletal muscle strength in other patient populations (e.g., the elderly, patients with cancer, patients with chronic obstructive pulmonary disease, COPD) and it is reasonable to believe that one or more of these agents may also be capable of increasing muscle strength in the mechanically ventilated MICU population [13–15]. There are, however, methodological issues that will need to be addressed to optimize performance of therapeutic trials of anabolic agents in critically ill mechanically ventilated patients. Most importantly, it will be useful if therapeutic trials incorporate an objective technique to accurately assess respiratory muscle strength.

There are two commonly used techniques that have been employed in the past to assess respiratory muscle strength in mechanically ventilated patients, i.e., the Pimax and the PdiTw in response to bilateral anterolateral magnetic phrenic nerve stimulation (BAMPS) [1–7, 16–18]. The Pimax has been severely criticized in the past as being unreliable in mechanically ventilated patients [7] and as a measure that does not discriminate between weaning success and weaning failure [3, 19]. In addition, there is no report, of which we are aware, that has shown that Pimax levels correlate with MICU patient survival. As a result, it could be argued that the Pimax should not be used to assess respiratory muscle strength in mechanically ventilated patients for clinical trials. If so, one could argue, future trials will be forced to employ techniques such as the PdiTw in response to BAMPS, to assess responses to therapies. Unfortunately, while the Pimax technique is simple, the PdiTw technique is complex and is only available in a handful of MICUs in the world. The purpose of the present study, therefore, was to compare measurements of Pimax and PdiTw in mechanically ventilated MICU patients to see how these two indices correlate with each other, and to ascertain the degree to which the Pimax, like the PdiTw, could predict outcomes in this patient population. To our knowledge, this is the largest MICU patient population in which these two measurements were made and were correlated with outcomes.

When comparing Pimax and PdiTw, it is important to first consider that there are several fundamental differences between these techniques. For one thing, the Pimax assesses the cooperative generation of inspiratory pressure by all the inspiratory muscles, while the PdiTw elicited by magnetic stimulation of the phrenic nerves assesses mainly the diaphragm. As a result, variation in the degree of rib cage activation during the Pimax maneuver can alter the level of Pimax for a given level of diaphragm strength and, by inference, a given level of PdiTw. In addition, the Pimax measures maximal voluntary inspiratory muscle pressure generation while the PdiTw assesses submaximal performance (i.e., pressure generation in response to a single neural impulse). Third, PdiTw levels are influenced by the previous contraction history of the diaphragm (i.e., a phenomenon termed twitch potentiation) while the Pimax is not [5, 6]. The findings in the present report underscore the potential for differences between Pimax and PdiTw measurements. In keeping with these factors, we found that there was substantial scatter when plotting Pimax against PdiTw, indicating that it is not possible to reliably predict PdiTw values from determination of Pimax alone.

The present data also provide the novel finding that infections systematically change the relationship between Pimax and PdiTw measurements; the present manuscript is the first to suggest this same phenomenon occurs in both animals and patients. Specifically, we found that infection selectively reduced skeletal muscle low-frequency force generation more than high-frequency force generation by comparing diaphragm force frequency curves for muscles obtained from control and mice treated with endotoxin. Similarly, we found that infected patients demonstrated a proportionately greater reduction of the PdiTw, an index of low-frequency force generation, than the Pimax, an index of high-frequency force generation, when compared to values for noninfected mechanically ventilated MICU patients. This greater reduction in low-frequency force generation may reflect the reported effect of infection to reduce sarcoplasmic reticulum calcium release and reuptake [20]. Another factor that may contribute to this phenomenon is the reported effect of infection to reduce the calcium sensitivity of skeletal muscle contractile proteins [21]. Regardless of the mechanism, this phenomenon results in a higher Pimax/PdiTw ratio for infected patients when compared to noninfected mechanically ventilated MICU patients.

It is also clear patients on mechanical ventilators have varying levels of respiratory drive due to differences in levels of lung disease, reflex sensitivity, ventilator settings, level of sedation, and other factors. In theory, lower levels of respiratory drive should depress the level of Pimax generated for a given level of intrinsic diaphragm force generating capacity, e.g., for a given level of PdiTw. As an extreme, Pimax could hypothetically be reduced to zero when extremely high levels of sedation are provided, even when PdiTw is very good. Consistent with this possibility, several patients had very low Pimax/PdiTw ratios in the present study (i.e., ratios as low as 1.54). Also in keeping with this concept, we found that patients with high levels of respiratory drive, i.e., that initiated (triggered) all breaths, had significantly higher Pimax/PdiTw ratios than patients with submaximal levels of ventilator triggering. Thus the dual influences of varying levels of infection and respiratory drive from patient to patient may well account for much of the variability in the Pimax to PdiTw relationship, with infection raising and low levels of respiratory drive lowering the Pimax for a given level of PdiTw.

Another factor that, in theory, may have contributed to the differences in PdiTw and Pimax measurements in the present study is related to the fact that we assessed PdiTw while patients continued on mechanical ventilation (to minimize twitch potentiation) while we assessed Pimax while patients were temporarily removed from mechanical ventilation (to facilitate recruitment of respiratory muscles during the maneuver). It is theoretically possible then that inspiratory muscle length may have changed, in some patients, when patients were transiently removed from mechanical ventilation in order to assess Pimax, further artifactually altering the PdiTw and Pimax relationship.

A final consideration when comparing these two indices, i.e., Pimax and PdiTw, are technical issues that may limit the accuracy of these assessments. As indicated above, inadequate levels of respiratory drive may result in artifactually low Pimax levels. There are several approaches to minimizing this artifact, including employment of prolonged airway occlusion (e.g., more than 20 seconds) for intubated patients, adding dead space to the respiratory circuit to increase respiratory drive, and ascertainment that the negative pressure generated at 100 milliseconds after initiation of an inspiratory effort (P0.1) is more negative than 2 cm H_2_O before determination of the Pimax [22]. For the PdiTw, attainment of supramaximal phrenic nerve stimulation during magnetic stimulation is necessary to ensure that all motor nerve fibers to diaphragm are maximally activated during this maneuver. Under laboratory conditions, proof of supramaximal phrenic nerve stimulation is best ascertained by repeatedly stimulating the phrenic nerves over a broad range of magnetic stimulation field strengths (from 60–100 % for the MagStim 200 unit) with multiple stimuli applied (e.g., more than five) at each level of field strength. The magnetic stimulation paradigm employed to obtain PdiTw data in the present manuscript represents a compromise between the need to attain perfect data and ethical constraints to minimize patient discomfort. While it is therefore possible that our phrenic stimulation technique may have underestimated PdiTw in some of our patients, previous studies suggest that supramaximal phrenic nerve stimulation can be achieved in the large majority of critically ill patients using the procedures employed in the present study [4, 5].

While the above discussion evaluates factors and artifactual issues that differentially influence Pimax and PdiTw determinations, a larger issue is which of these two indices is a better index of physiological function and which one is a better predictor of patient outcomes. Which is the more relevant physiologically may well depend upon the specific respiratory task that a patient may encounter. Even in the presence of lung disease, the diaphragm does not normally generate “ballistic” repetitive contractions at near maximal levels of force generation (and extremely high levels of stimulation frequencies) during breathing efforts but is driven in response to mean stimulation frequencies in the 5–15 Hz range. In theory, therefore, diaphragm force generation in response to relatively low stimulation frequencies (closer to twitch forces) may be a more relevant index of diaphragm performance during normal breathing than diaphragm force output in response to maximal levels of excitation (i.e., Pimax of MIP maneuvers). On the other hand, ballistic efforts, such as coughing or sneezing, involve high-level activation of both inspiratory and expiratory muscles. During such maneuvers, the Pimax may well be a better index of predictor of task performance.

An even more important issue, however, is which parameter (Pimax or PdiTw) is a better predictor of patient outcomes. Arguably, the two most important outcomes for critically ill mechanically ventilated ICU patients are whether they survive, and if they survive, how long it takes to wean them from mechanical ventilation. Our data indicate that PdiTw is a good predictor of both mortality and duration of mechanical ventilation. Surprisingly, we also found that Pimax also was a fair predictor of these two outcomes, with patients with lower Pimax levels having a statistically greater mortality and, if they survived, a longer duration of mechanical ventilation than patients with high Pimax levels. While both indices were statistically predictive of mortality and mechanical ventilation duration, PdiTw was the better predictor, since this index had higher F scores than the Pimax for correlation with both outcomes.

This analysis also raises the question as to why PdiTw level was the better predictor of clinical outcomes. This may result because there is more biological variability in Pimax than the PdiTw due to the factors listed above (infection, alterations in sedation, variation in respiratory drive) so that it is more difficult to distinguish the true level of weakness using Pimax than using PdiTw measurements. PdiTw is also a better measure of diaphragm function at low frequencies of neural activation. As suggested above, since the mean endogenous phrenic neuron firing frequency is relatively low [23], it is possible that the PdiTw may be a more relevant index of the ability of the diaphragm to function close to the range of firing frequencies that are physiologically achieved. In addition, while the other inspiratory muscles clearly contribute to breathing in patients with respiratory failure, the diaphragm is the major muscle of respiration and diaphragm strength per se may be a better determinant of outcomes.

An even more fundamental issue raised by our data is whether or not the observed correlation between respiratory muscle strength and mortality might be influenced by other factors that may have contributed to death in our patient population. To further analyze this issue, we performed a series of multivariate analyses examining the impact of several potentially confounding variables (namely, sepsis, age, gender, steroid usage, respiratory system static compliance, inspiratory airway resistance, SOFA score, and comorbidity score) on the relationship between weakness and mortality. As indicated by our results (Tables 2 and 3), age, gender, steroid usage, respiratory system static compliance, and inspiratory airway resistance were not significantly correlated with mortality in any of our statistical analyses, while our two indices of respiratory muscle strength (PdiTw and Pimax) were highly significant correlates of survival in all analyses. When included in this multivariate analysis, the presence and absence of sepsis was also not significantly correlated with mortality. Finally, when we statistically analyzed only the subgroup of patients in our study that were septic, respiratory muscle weakness remained, by far, the best predictor of mortality (Tables 4 and 5).

We should consider the potential mechanism(s) by which diaphragm weakness may have influenced mortality. One of the patients that died in our study had a large stroke, never regained consciousness, and died when his family withdrew care. An additional five patients were in shock at the time of death. It is therefore difficult to implicate weakness as a direct contributor to death in these six patients. In the remaining 12 patients who died in our study, however, none were in shock, all had good Glasgow coma scores, all were weak, and in all 12 it was not possible to successfully wean these patients from mechanical ventilatory support. In all 12, death occurred once the patients’ families decided to withdraw life support, and the only form of life support that these 12 patients were receiving and that was withdrawn was mechanical ventilation. Importantly, the lung function of these 12 patients was not different, on average, from patients that survived (also, lung function was not statistically associated with death in our multivariate analysis). Based on this analysis, we believe that it is reasonable to speculate that it is possible that diaphragm weakness may contribute to death by preventing weaning of patients from mechanical ventilation, thereby contributing to a decision to withdraw life support. The only way to conclusively prove this point, however, is to perform prospective studies to determine if therapies that increase diaphragm strength reduce mortality in mechanically ventilated ICU patients.

## Conclusions

In summary, our data indicate that both Pimax and PdiTw levels are both extremely low in the average mechanically ventilated patient. There was a fair correlation between these two indices for our patient population as a whole, with Pimax, on average, equal to approximately 4.5 times the PdiTw. For individual patients, however, there is substantial variation in the Pimax/PdiTw ratio, such that individual patient PdiTw levels cannot be reliably estimated based on the Pimax level alone. Several factors appear to influence the Pimax/PdiTw ratio, with infection inducing an increase in this ratio and reductions in respiratory motor output, as judged by the degree of ventilator triggering, producing a reduction in the Pimax/PdiTw ratio. PdiTw was an excellent predictor of both patient survival and the time required to wean patients from mechanical ventilation. Surprisingly, Pimax was also a predictor of survival and a fair predictor of mechanical ventilation duration. Whether PdiTw or Pimax should be employed to assess diaphragm strength in MICU patients may depend upon assessment goals. Arguably, in research settings requiring an index of respiratory muscle strength that is an excellent predictor of patient outcomes, the PdiTw measurement may be the better technique. In clinical settings requiring an easily performed test with more limited goals (e.g., to screen for possible neuromuscular disease), the Pimax appears to provide clinically relevant information if properly performed.

## Key messages

- The present study found that respiratory muscle strength, measured either by assessment of Pimax or PdiTw measurements, was profoundly reduced in mechanically ventilated MICU patients.
- We also found that PdiTw correlated with Pimax values (r^2^ = 0.373, *p* < 0.001) but there was wide scatter for this relationship and PdiTw could not be reliably calculated from Pimax levels for individual subjects.
- Two clinical factors systematically affected the PdiTw/Pimax ratio: (a) the presence of serious infection significantly reduced this ratio, and (b) reductions in respiratory drive increase this ratio.
- These data indicate that PdiTw is an excellent predictor of both patient survival and the time required to wean patients from mechanical ventilation.
- These data also show that Pimax correlates with both survival and duration of mechanical ventilation, but is statistically is a weaker predictor than PdiTw

## Additional file

Additional file 1: Methods: Assessment of mechanical ventilator triggering, Measurement of respiratory system static compliance and airway resistance, Determination of transdiaphragmatic twitch pressure generation (PdiTw), Determination of maximum inspiratory pressure (Pimax). (DOCX 17 kb)

## Abbreviations

BAMPS: bilateral anterolateral magnetic phrenic nerve stimulation
FiO_2_: fraction of inspired oxygen
H_2_O: water
ICU: intensive care unit
LPS: lipopolysaccharide
MICU: medical intensive care unit
P0.1: inspiratory pressure at 100 milliseconds after initiation of a breath
PdiTw: transdiaphragmatic twitch pressure in response to bilateral magnetic stimulation of the phrenic nerves
PesoTw: esophageal pressure generated during the twitch assessment
Pimax: maximum inspiratory pressure
SOFA: Sequential Organ Failure Assessment
