# Supplementary material for: Correlation of maximal inspiratory pressure to transdiaphragmatic twitch pressure in intensive care unit patients
Source: Crit Care. 2016 Mar 23;20:77. doi: 10.1186/s13054-016-1247-z (PMC4818524; doi:10.1186/s13054-016-1247-z)
Supplement: Additional file 1: — Methods: Assessment of mechanical ventilator triggering, Measurement of respiratory system static compliance and airway resistance, Determination of transdiaphragmatic twitch pressure generation (PdiTw), Determination of maximum inspiratory pressure (Pimax). (DOCX 17 kb) [file 13054_2016_1247_MOESM1_ESM.docx]

**Additional file 1**

**Assessment of Mechanical Ventilator Triggering**

We assessed ventilator triggering prior to all other physiological measurements. This assessment was made after the patient was allowed to quietly breathe on the ventilator settings prescribed by their ICU care team for a minimum of 15 minutes. All of the patients included in the current data set were on a full support ventilator mode at the time of study (e.g. assist/control, pressure control) and none were on “weaning” modes of ventilation (e.g. pressure support). All patients were ventilated using a Siemens ventilator, which detects inspiratory efforts by assessing alterations in the airway flow and pressure that are initiated by patient efforts. We recorded the number of times the ventilator delivered “triggered” breaths out of the total number of breaths delivered over a two minute interval and this fraction is reported as % breath triggering, For all studies, patients were on the same ventilator mode and settings during the period of assessment of ventilator triggering as that provided during the period during which PdiTw measurements were later made.

**Measurement of Respiratory System Static Compliance and Airway Resistance**

For this assessment, the ventilator was set to a square-wave flow pattern with an inspiratory plateau. Ventilator rate was transiently increased (e.g. 30-90 seconds) to suppress spontaneous respirations. Peak pressure (Ppeak) and plateau pressure (Pplat) were recorded and intrinsic PEEP (PEEPi) was determined using an end-expiratory occlusion. The inspiratory airway resistance of the respiratory system was calculated as (Ppeak − Pplat)/inspiratory flow, and the effective static respiratory system compliance was calculated as Vt/(Pplat – (total PEEP)). Once static maneuvers were completed, the ventilator frequency was returned to its previous level. Static measurements were made before determination of PdiTw and Pimax.

**Determination of Transdiaphragmatic Twitch Pressure Generation (PdiTw)**

Diaphragm strength was assessed by determining transdiaphragmatic twitch pressure (PdiTw) in response to bilateral anterior magnetic stimulation of the phrenic nerves (BAMPS) as previously described [1]. All subjects were studied in the supine position with the head of the bed elevated at 30 degrees. Two sterile balloon tipped catheters (Ackred Medical, NJ) were passed through the nose after application of local anesthetic (1 cc of 1% Lidocaine gel); one catheter was placed in the stomach and the other was placed in the esophagus. Correct gastric balloon placement was confirmed by demonstrating a positive pressure in response to pressure applied over the stomach. Correct esophageal balloon placement was verified by demonstrating that the pressure waveform tracked airway pressure changes (i.e. the two values were within 20%) with inspiratory efforts during airway occlusion.

After balloon placement, subjects were allowed to breathe quietly for 20 minutes before further assessment. This delay was provided to minimize the possibility of twitch potentiation (a transient increase in the amplitude of the twitch when it is preceded by a forceful contraction). Figure of eight magnetic coils attached to dual Magstim 200 stimulators (Jali Medical, Inc., Waltham, MA,) were subsequently placed bilaterally over the phrenic nerves. Simultaneous magnetic pulses were delivered to the phrenic nerves bilaterally to elicit twitch transdiaphragmatic pressures (i.e. PdiTw), while transiently occluding the external circuit connecting the endotracheal tube to the ventilator with a pneumatic valve. All stimulations were timed to occur at end-expiration in the ventilatory cycle. A minimum of five twitches were recorded at 100% magnetic field strength, with at least 30 seconds between adjacent stimuli. To verify stimuli were supramaximal, multiple additional twitches (3-8) were performed at reduced magnetic field strengths (80-95%) and 100% field strength was operationally defined to be supramaximal if this value was similar to twitch forces obtained at lower magnetic field strengths (e.g. identical to twitch pressures at 90% and 95% field levels).

Analysis of twitches was accomplished by measuring twitch esophageal pressure and twitch gastric pressure as the difference between the pressures immediately before phrenic nerve stimulation and the peak pressures achieved following phrenic activation. Individual twitches were considered acceptable for analysis if there was a consistent esophageal pressure immediately prior to twitch determination, if there was no esophageal peristalsis altering the esophageal pressure tracing and if no evidence of coughing was present. The best three measurements in response to 100% levels of magnetic stimulation were averaged and recorded as the PdiTw.

**Determination of Maximum Inspiratory Pressure (Pimax)**

To determine maximum inspiratory pressure (Pimax), the ventilator circuit was detached from the endotracheal tube and replaced with a plastic tube containing one way valves to separate inspiratory and expiratory airflow (NIF-Tee®, Smith Medical, Keane, NH). A port in the NIF-Tee® valve was then connected to a Validyne pressure transducer using plastic tubing. The inspiratory limb of the NIF-Tee® valve was occluded and patients were then encouraged to breathe in repeatedly as hard as they could. The circuit was kept occluded for at least 30 seconds but no more than 60 seconds, with monitoring of the pulse oximeter to ensure that patients did not become hypoxic during the measurements. Subjects made at least 5 inspiratory efforts during the inspiratory occlusion trial. The three best pressures were averaged and taken as the Pimax.

**In Vitro Determination of Animal Diaphragm Force Generation**

Diaphragm strips of approximately 3-5 mg in size were dissected from the left costal diaphragm and mounted in a temperature controlled jacketed bath containing curarized Krebs-Henseleit solution as previously described [2]. The central tendon end of strips was connected to a force transducer above the bath (Scientific Instruments, Heidelberg, Germany). Muscle stimulation was accomplished by delivering current from a constant amperage amplifier driven by a Grass 48 stimulator (Grass, West Warwick, RI, USA) to platinum filed electrodes surrounding the diaphragm strip. After a 15 minute equilibration period, diaphragm strips were adjusted to the length providing maximum tension and stimulation current was adjusted to supramaximal levels. Strips were then stimulated to contract in response to trains of 1, 20, 50,100 and 150 Hz, with each train lasting 500 ms and with a 30 second rest between adjacent trains. Force output was recorded graphically using a Kipp-Zonen recorder (Bohemia, NY, USA). Strip length was measured using a micrometer. Muscle cross sectional area (CSA) was calculated using the formula of Close (i.e. muscle weight times density (1.06) divided by length) [3] . Muscle specific force was calculated as force divided by cross sectional area.

**REFERENCES**

1. Supinski GS, Callahan LA: **Diaphragm weakness in mechanically ventilated critically ill patients**. *Crit Care* 2013, **17**(3):R120.

2. Supinski GS, Ji X, Callahan LA: **The JNK MAP kinase pathway contributes to the development of endotoxin-induced diaphragm caspase activation**. *Am J Physiol Regul Integr Comp Physiol* 2009, **297**(3):R825-834.

3. Close RI: **Dynamic properties of mammalian skeletal muscles**. *Physiol Rev* 1972, **52**(1):129-197.
